# Mucoadhesive and Antimicrobial Allantoin/β Cyclodextrins-Loaded Carbopol Gels as Scaffolds for Regenerative Medicine

**DOI:** 10.3390/gels8070416

**Published:** 2022-07-02

**Authors:** Daniela Filip, Doina Macocinschi, Mirela-Fernanda Zaltariov, Carmen Anatolia Gafitanu, Cristina Gabriela Tuchilus, Adrian Bele, Bianca-Iulia Ciubotaru, Elena Stoleru, Alexandra Bargan

**Affiliations:** 1 “Petru Poni” Institute of Macromolecular Chemistry, Aleea Gr. Ghica Voda 41 A, 700487 Iasi, Romania; dare67ro@yahoo.com (D.F.); bele.adrian@icmpp.ro (A.B.); ciubotaru.bianca@icmpp.ro (B.-I.C.); elena.paslaru@icmpp.ro (E.S.); anistor@icmpp.ro (A.B.); 2 Department of Pharmaceutical Technology, Faculty of Pharmacy, “Gr. T. Popa” University of Medicine and Pharmacy, 16 Universitatii Street, 700115 Iasi, Romania; carmengafitanu@gmail.com; 3 “Microbiology Department, Faculty of Medicine, “Gr. T. Popa” University of Medicine and Pharmacy, 16 Universitatii Street, 700115 Iasi, Romania; ctuchilus@yahoo.com

**Keywords:** β-cyclodextrin/allantoin inclusion complexes, interfacial energy, mucoadhesion, antibacterial, tissue engineering scaffolds

## Abstract

Allantoin and its β-cyclodextrin and hydroxypropyl-β-cyclodextrin inclusion complexes 1:1 have been used to prepare carbopol-based mucoadhesive gels. The gelation process occurred by adjustment with glycerol 10% in the presence of triethanolamine. The structural features induced by the presence of allantoin and the corresponding β-cyclodextrins inclusion complexes have been first investigated by infrared spectroscopy highlighting strong interactions within the gels network and ideal crosslinks for the self-healing behavior. The hydrophilicity of the gels was investigated by the determination of the surface tension parameters and the free energy of hydration. The interfacial free energy values indicated prolonged biocompatibility with blood. The gels-water molecule interactions in terms of sorption, permeability, and diffusion coefficients were evaluated by dynamic vapor sorption analysis. The self-assembly process through intermolecular H-bonding, the high hydrophilicity, the mechanical performance, the hydrolytic stability in simulated biological media, the biocompatibility with normal human dermal fibroblast (NHDF) cells, the mucoadhesivity and antimicrobial activity on selected microorganism species (*S. Aureus* and *C. albicans*) of the allantoin-based gels recommend them as promising scaffold alternatives in regenerative medicine.

## 1. Introduction

Tissue engineering is an interdisciplinary domain in medicine that combines the benefits of different areas from chemistry, physics and chemistry, materials science, and engineering. The main concern of this field is the development of new biological substitutes/scaffolds able to improve or restore the damaged tissues or/and organ functionalities by their own physical, chemical, and mechanical properties. To date, there are some approaches, such as allogenic transplants and artificial implants, with a series of disadvantages related to the finding of suitable donors, the post-operative complications, and the most, referring to the transplant rejection [1].

In this context, the finding of high biocompatible and biodegradable materials able to sustain the proliferation and the differentiation of functionalized cells as well as their interactions and also offer beneficial mechanical support became a special opportunity to develop new scaffolds for regenerative medicine. Such biomaterials proved to be hydrogels, physically or chemically crosslinking 3D structures that mimic a 3D cellular microenvironment, by their biophysical properties: higher water content, viscoelasticity, mechanical strength, and self-healing ability. Moreover, hydrogels are ideal candidates for combined cell culture and drug delivery systems, most of them providing similar structures to those of extracellular matrix, thus improving biocompatibility and the specific cellular response in contact with them by reducing the incidence of inflammatory, immunological, and toxicity side effects [1,2].

These materials are also successfully designed for the dressing of skin wounds due to their high oxygen permeability and suitable wettability necessary for applications in soft tissues. The most recognized polymers for gel applications are carbomers, cellulose derivatives, polyvinyl alcohol, and hyaluronic acid, which are biocompatible and bioadhesive, showing suitable viscoelastic behavior and thermal stability, as well as suitable acceptance when implanted [3].

Carbopol or polyacrylic acid (PAA) can be easily crosslinked in 3D hydrogels with a high swelling ability in neutral form. It is used in pharmaceutical excipients or as nanogels acting as drug carriers with improved mucoadhesiveness, enhancing the drug’s bioavailability [4], in the regeneration of bone tissues [5], as 3D scaffolds for cell cultures [6,7], in wound healing due to its low viscosity, high transparency and a suitable ion tolerance, providing a moist medium, helping the elimination of exudates, preventing the infections, thus promoting a suitable device for tissue regeneration [8,9,10] and in numerous topical drug delivery systems [11,12].

Thus, the drug permeation can be a real challenge that can be controlled by the design of scaffolds and dosage forms according to the local physiological factors to ensure the permeability through the local environment of the tissue mucosa. Based upon the potential site of absorption/application and adhesive strength, polymer characteristics, biocompatibility, and safety, suitable mucoadhesive polymer scaffolds should be selected, and preparation techniques should be adopted. The various mucoadhesive polymers used for the development and functionalization of regenerative medicine also include cyanoacrylates, polycarbophil, chitosan, and gellan and their blends with different natural and synthetic polymers [13,14,15].

Cyclodextrins are oligosaccharides that have been used extensively as pharmaceutical excipients. Because of their ability to form inclusion complexes with hydrophobic drug molecules, their main applications refer mainly to the improvement in the solubility and the dissolution of drugs [16,17,18].

Cyclodextrins behave as “host” molecules due to their particular structure, hydrophilic to the outside and lipophilic at the inner cavity level. They totally or partially include the active substance molecule, called “guest”, in the hydrophobic cavity. From the formed inclusion complex, cyclodextrins release the active principle in the administration site, improving bioavailability.

In order to carry out the research, there was used β-cyclodextrin and hydroxypropyl-β-cyclodextrin with an inner cavity diameter large enough to include allantoin with its fixation by means of sufficiently strong bonds, which makes it possible to obtain stable complexes. Compared to β-cyclodextrin, hydroxypropyl-β-cyclodextrin has the advantage that, by substituting on OH groups, there are changes in hydrogen bonds, which results in an increase in water solubility. Allantoin isolated from *Symphytum off*. is a poor-soluble drug in water. Allantoin has anti-inflammatory properties, as well as anti-irritant and keratolytic effects, being used in varied dermatological and cosmetic/pharmaceutical products at a concentration of 0.1–2%, proving to be nontoxic with a less of side effects: irritating or allergic ones [19,20].

In this study, allantoin loaded with β-cyclodextrin and hydroxypropyl-β-cyclodextrin, respectively, in 1:1 ratio-based bioadhesive gels have been obtained. Carbopol 934 is the bioadhesive polymer used to prepare biocompatible and antimicrobial mucoadhesive gels. The carbopol hydrogels are physically stable at the physiological pH of the body, and they maintain their rheological properties and semi-solid consistency and also contribute to a gradual release of allantoin, avoiding the local tissue infections during the healing process when it is used in tissue engineering applications.

## 2. Materials and Methods

### 2.1. Materials

The materials used in this study were purchased and used as received: allantoin (Elton Chemical SA, Avlonas, Greece), β-cyclodextrin (β-CD) (Fluka, Ph. Eur.), hydroxypropyl-β-cyclodextrin (HP-β-CD) (Fluka, Ph. Eur.), Carbopol 934 or poly(acrylic acid), (PAA) (ServaFeinBiochemica, Heidelberg, Germany), triethanolamine (TEA) (Sigma-Aldrich, Schnelldorf, Germany), glycerol (CREMER OLEO, Hamburg, Germany), distilled water (FRX), NHDF (Normal Human Dermal Fibroblast) cell line from PromoCell, complete cell medium containing Eagle’s Minimal Essential Medium alpha, aMEM, Dulbecco’s Modified Eagle Medium without phenol red (DMEM), a mixture of penicillin-streptomycin-amphotericin B mixture of concentration of 1% (10 K/10 K/25 μg in 100 mL), EDTA (Trypsin-Versene mixture from Lonza), Fetal Bovine Serum (FBS) achieved from Biochrom GmbH (Berlin, Germany) and Phosphate Buffered Saline (PBS) from Invitrogen.

### 2.2. Preparation of the Mucoadhesive Gels

Carbopol 934 (0.5% *w*/*w* and 1% *w*/*w*) was added to a mixture of water/glycerol and stirred at room temperature until a homogeneous dispersion. Then, a few drops of TEA were added, and the obtained transparent gel solutions were left overnight for complete hydration. The measured pH of the gels was 6.5, and the final mass was adjusted by the addition of water (up to 100 g). Allantoin (0.2% *w*/*w*) was used as such and in the composition of the inclusion complexes 1:1 ratio with β-cyclodextrin and hydroxypropyl-β-cyclodextrin, respectively, and added to the initial gel mixture. The prepared samples (Table 1) were frozen in liquid nitrogen and then freeze-dried.

### 2.3. Methods

FT-IR spectra were recorded using an FT-IR Bruker Vertex 70 (Ettlingen, Germany) spectrometer equipped with a ZnSe crystal. The spectra were recorded in the 600–4000 cm^−1^ wavenumber range in attenuated total reflectance (ATR) mode at room temperature with 32 scans per spectrum at a resolution of 4 cm^−1^. The hydrogen-bonded distances (R) and the hydrogen bond energy (*E_H_*) were calculated by using Pimentel and Sederholm Equations (1) and (2) [21,22]:

(1) $$E_{H}=\frac{1}{k}\times \frac{\nu_{0}-\nu }{\nu_{0}}$$

Δ*ν* = 4.43 × 10^3^ × (2.84 − *R*) (2)

where Δ*ν* = *ν* − *ν*_0_, *ν*_0_ is the monomeric O-H stretching frequency located at 3650 cm^−1^, *ν* is the stretching frequency of the H-bonded O-H groups in the IR spectra of the analyzed samples, *k* is a constant (1/*k* = 2.625 × 10^2^ kJ).

^1^H NMR spectra were registered on a Bruker Advance NEO 400 MHz Spectrometer equipped with a 5 mm QNP direct detection probe and z-gradients. The spectra were recorded in D_2_O at room temperature, and the chemical shifts are reported as δ values (ppm) using the solvent residual peak as reference.

Martin Christ ALPHA 1-2LD lyophilizer (48 h at −57 °C and 0.045 mbar) was used to freeze-dry the samples.

Static contact angles were determined by the sessile drop method, using a CAM-200 instrument from KSV (Helsinki, Finland), by placing a 1 μL drop of water on the film surface and waiting for 2 s before recording. Drop shape was recorded with a high-speed framing camera; the images were then processed by a computer and stored. Contact angle measurements were taken at least 6 times at different locations on the surface, and the average values were considered for further analysis. The determinations were made at room temperature (23 °C and 40% relative humidity). The components of the surface tension of the polymer gels have been estimated based on the contact angles at equilibrium between the gel surface and three standard liquids with high purity: twice-distilled water, formamide, and methylene iodide (CH_2_I_2_). The Young–Laplace equation was applied to study the drop profile of the samples.

The formulas used in calculating surface tension parameters are based on the geometric mean method, Equations (3) and (4), and the acid/base method (*LW*/*AB*), Equations (5)–(7) [23,24,25,26,27]:

(3) $$\frac{1+\cos\theta }{2}\frac{\gamma_{lv}}{\sqrt{\gamma_{lv}^{d}}}=\sqrt{\gamma_{sv}^{p}}\cdot \sqrt{\frac{\gamma_{lv}^{p}}{\gamma_{lv}^{d}}}+\sqrt{\gamma_{sv}^{d}}$$

(4) $$\gamma_{sv}=\gamma_{sv}^{d}+\gamma_{sv}^{p}$$

where *θ* is the contact angle determined for triplet water, formamide, and CH_2_I_2_, subscripts “*lv*” and “*sv*” describe the interfacial liquid–vapor and surface–vapor tensions, respectively, while superscripts “*p*” and “*d*” describe the “polar” and the “disperse” components, respectively, of total surface tension, *γ_sv_*.

(5) $$1+\cos\theta =\frac{2}{\gamma_{lv}}(\sqrt{\gamma_{sv}^{LW}\cdot \gamma_{lv}^{LW}}+\sqrt{\gamma_{sv}^{+}\cdot \gamma_{lv}^{-}}+\sqrt{\gamma_{sv}^{-}\cdot \gamma_{lv}^{+}}$$

(6) $$\gamma_{sv}^{AB}=2\sqrt{\gamma_{sv}^{+}\cdot \gamma_{sv}^{-}}$$

(7) $$\gamma_{sv}^{LW/AB}=\gamma_{sv}^{LW}+\gamma_{sv}^{AB}$$

where superscripts “*LW*” and “*AB*” are the disperse and the polar component obtained from the $\gamma_{sv}^{-}$ electron donor (Lewis base) and the $\gamma_{sv}^{+}$ electron acceptor (Lewis acid) interactions, while superscript “*LW*/*AB*” is the total surface tension.

The solid surface tension components were evaluated by means of the geometric mean method Equation (3) [28], using the known surface tension components of tested liquids and the contact angles from Table 2. The total surface tension was calculated with Equation (4).

The hydrophilicity of solid surfaces was estimated with respect to the free energy of hydration, Δ*G_w_*. (Equation (8)):

Δ*G_w_* = −*γ_lv_*(1 + cos *θ_water_*) (8)

where *γ_lv_* is the surface energy of liquid water and *θ_water_* is the contact angle of water with dried formulations.

Solid–liquid interfacial tension, $\gamma_{sl}$, is given [29] by the following equation:

(9) $$\gamma_{sl}={\left(\sqrt{\gamma_{lv}^{p}}-\sqrt{\gamma_{sv}^{p}}\right)}^{2}+{\left(\sqrt{\gamma_{lv}^{d}}-\sqrt{\gamma_{sv}^{d}}\right)}^{2}$$

$\gamma_{sl}$ for the dried formulations were evaluated both for water and human blood ($\gamma^{d}$ = 11.2 mN/m and $\gamma^{p}$ = 36.3 mN/m) [30].

The spreading coefficient, *S_C_*, measures the tendency of the liquid phase to spread (complete wetting) on the solid phase and is given by:

(10) $$S_{C}=\gamma_{sv}-\gamma_{sl}-\gamma_{lv}$$

When *S_C_* is positive, spontaneous wetting occurs, but when *S_C_* is negative, the liquid will not spread spontaneously over the solid substrate. The degree of interaction between the test liquids and dried formulations can be described by Girifalco–Good’s interaction parameter, *Φ*, which represents a characteristic of the system that can be estimated from the molecular properties of the two phases (Equation (11)):

(11) $$\Phi =\frac{\gamma_{lv}(1+\cos\theta )}{2{\left(\gamma_{lv}\gamma_{sv}\right)}^{1/2}}$$

Work of adhesion represents the work required to separate the liquid and solid phases and describes the strength of the interaction between the two phases (Equation (12)):

(12) $$W_{a}=\gamma_{lv}(1+\cos\theta )$$

When the work of adhesion is positive, the surfaces will bond, and by increasing the strength of the bond, higher values are obtained. For negative values, no bonding will come about. Blood-contacting devices require a suitable balance of hydrophilic and hydrophobic surface entities because excessively hydrophobic surfaces enhance cell affinity and reduce biocompatibility, but highly hydrophilic surfaces prevent cell–cell interactions, which are particularly important in tissue engineering [31].

Dynamic vapor sorption analysis was performed by using IGAsorp equipment (Hiden Analytical, Warrington, UK) in the relative humidity range (HR) 0–90%, at 25 °C. The equipment includes an analytical microbalance through the initial mass and the weight changes induced by the increase in humidity, which can be determined with great precision. Before starting the experiments, the samples were dried in flowing nitrogen (250 mL/min) at 25 °C. To determine the water sorption capacity of the sample, the relative humidity was changed in stages from 10% each up to 90%. The pre-established equilibrium for each stage was 50–60 min.

The sorption coefficient, *S*, is a thermodynamic parameter that depends on the polymer-water molecules interactions and measures the extent of sorption. The sorption coefficient is given by Equation (13) [32]:

(13) $$S=\frac{M_{\infty }}{M_{0}}$$

where *M_∞_* is the mass of water taken up at equilibrium and *M*_0_ represents the initial mass of the polymer sample.

The permeability coefficient, *P* [33], refers to the water permeated through the uniform area of the sample per second and is given by the product of diffusion and sorption coefficients as in Equation (14):

(14) P=D×S

Blood compatibility is dictated by the manner in which their surfaces interact with blood constituents, such as red blood cells and platelets. The measurement of the surface and interfacial free energy of a material constitutes an in vitro method for determining biocompatibility. For establishing the material’s compatibility with blood, Equation (15) was used, where *W_s/rbc_* and *W_s/p_* describe the work of spreading red blood cells and platelets [34].

(15) $$W_{s}=W_{a}-W_{c}=2\left(\sqrt{\gamma_{sv}^{LW}\gamma_{lv}^{LW}}+\sqrt{\gamma_{sv}^{+}\gamma_{lv}^{-}}+\sqrt{\gamma_{sv}^{-}\gamma_{lv}^{+}}\right)-2\gamma_{lv}$$

where *W_s_*—work of spreading (the negative free energy associated with spreading liquid over the solid surface); *W_a_*—work of adhesion (defined as the work required to separate the liquid and solid phases) and *W_c_*—work of cohesion (defined as the work required to separate a liquid into two parts).

The diffusion coefficient (*D*) can be obtained from a plot giving the ratio of the swollen polymer mass at time *t* and *t* = *∞* (corresponding to sorption equilibrium), the initial slope of a plot of *M_t_*/*M_∞_* as a function of the square root of time *t*^1/2^ or limiting slope of a plot of *ln*(1 − *M_t_*/*M_∞_*) vs. *t*. At sufficiently *short* times, it becomes determinant as in Equation (15):

(16) $$\frac{M_{t}}{M_{\infty }}=\frac{4}{l}\cdot \sqrt{\frac{D_{1}\cdot t}{\pi }}$$

so: (*M_t_*/*M_∞_*)^2^ = 16·*D*_1_·*t*/*π*·*l*^2^ = *K*_1_·*t*, where: *K*_1_ = 16·*D*_1_/*π*·*l*^2^, result: *D*_1_ = *K*_1_*πl*^2^/16.

At sufficiently *long* times, it becomes determinant as in Equation (16):

(17) $$\frac{M_{t}}{M_{\infty }}=1-\frac{8}{\pi^{2}}\cdot e^{-\frac{D_{2}\pi^{2}t}{l^{2}}}$$

so: *ln*(1 − *M_t_*/*M_∞_*) = *ln*8/*π*^2^ − *D*_2_·*π*^2^·*t*/*l*^2^ = *K*_2_·*t*, where: *K*_2_ = −*D*_2_·*π*^2^/*l*^2^, result: *D*_2_ = −*K*_2_*l*^2^/*π*^2^.

The mechanical tests have been performed by using an Instron 3365, a two-column universal mechanical testing device. The stress–strain measurements were made at an extension rate of 50 mm/min at room temperature (laboratory conditions). The compression tests were performed at 50 mm/min compression rate and 20% compressive strain.

#### 2.3.1. In Vitro Degradability of the Gels

The in vitro degradability of the gels was evaluated in conditions that mimic the body in a complete cell medium based on alpha-MEM (Eagle’s Minimal Essential Medium alpha), FBS (fetal bovine serum), and a 1% mixture of penicillin-streptomycin-amphotericin B at 37 °C. The degradability of the gels has been investigated by the FT-IR spectroscopy spectra subtraction function in OPUS 6.5 software. This procedure highlighted the changes occurring in gels during the hydrolysis process by comparing the initial spectra of the samples with those resulting after 24 h, 48 h, and 72 h immersion in the above-mentioned medium.

#### 2.3.2. Cell Culture and MTS Assay

NHDF cells were cultured first in aMEM medium containing 10% FBS and 1% penicillin-streptomycin-amphotericin B mixture in tissue culture flasks under a 5% CO_2_ humidified atmosphere at 37 °C. For passaging cells, Tryple was used for NHDF cells. The gels (30 mg of each sample) were introduced in 0.5 mL of a complete medium. Prior MTS assay, the NHDF cells were seeded on 96-well plates at densities of 5 × 10^3^ per well and incubated for 24 h. The next day the medium was replaced with a gel mixture of cell medium. Control wells were also prepared. Cells with gels were incubated for 48 h. At the end of 48 h, the cell medium was replaced with 100 µL/well DMEM without phenol red and 20 µL/well MTS reagent, and plates were again incubated for 3 h. The absorbance of formazan as a by-product of mitochondrial activity was estimated by using an iMark plate reader (Bio-Rad, Bucharest, Romania) at 490 nm.

The relative cell viability was calculated by the formula: V = 100 × (S − B)/(C − B), where V is the relative viability, S is the absorbance of the sample, B is the absorbance of the blanks, and C is the absorbance of the control samples.

Bio- and mucoadhesion tests were performed using a TA.XT Plus ^®^texture analyzer (Stable Micro Systems, UK) in order to measure the adhesion ability of the gels to cellulose membrane (cellulose dialysis tubing 12,000 Da) and porcine stomach mucosa, respectively. The material samples used were cut to the same dimension as the cylinder device, and the cellulose membrane was pre-boiled and cooled before being cut to 4 cm^2^ pieces in order to fit the holding device. Over each of the cellulose membrane or stomach mucosa samples, 200 µL of Phosphate Buffered Saline solution of pH 7.4 and 2.6, respectively, were added for the simulation of the physiological environment from the oral cavity and stomach, respectively. In addition, for the accurate simulation of the physiological environment, the holding device with the cellulose membrane, over which the cylinder with the material was lowered to make contact, was placed in heated distilled water to 37 °C and stirred at a speed of 250 rotations per minute. The cylindrical device with the material sample was lowered with a pre-determined speed of 1 mm/s to reach the cellulose membrane with a determined contact force of 9.80665 mN and for the pre-determined time of 30 s. The maximum detachment force and the work of adhesion parameters were determined using the TA.XT Plus^®^ texture analyzer software.

Antimicrobial activity was investigated by using the disc-diffusion methods [35,36] on Gram-positive (*Staphylococcus aureus* ATCC 25923) and Gram-negative (*Escherichia coli* ATCC 25922, *Pseudomonas aeruginosa* ATCC 27853) bacteria and one species of fungi (*Candida albicans* ATCC 10231). The microorganism suspensions were inoculated in a medium consisting of Mueller–Hinton agar (Oxoid) and Mueller–Hinton agar fungi (Biolab) in Petri plates. The tested compounds (100 µL) were added to the sterile stainless steel cylinders (5 mm internal diameter; 10 mm height), applied on the agar surface in the Petri plates, and left for a homogeneous diffusion in the medium for a period of 10 min at room temperature. The incubation of the samples was made at 37 °C for 24 h, after which the diameters of inhibition were measured. The tests have been performed in triplicate.

## 3. Results and Discussion

### 3.1. Structural Characterization by FT-IR Analysis

FT-IR spectroscopy identifies the vibrational patterns of guest-host molecules, the modifications in the characteristic bands such as disappearance, widening, deviations in their wavenumbers, or intensity changes.

**Figure 1:**
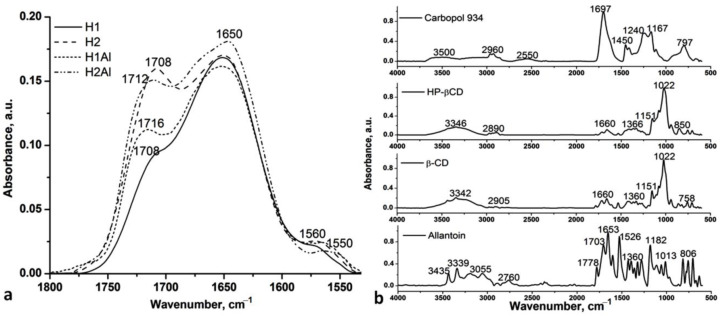
Carbonyl and amide II spectral regions of H1, H2, H1Al, and H2Al samples (**a**) and the comparative spectra of allantoin, β-CD, HP-β-CD, and Carbopol 934 (**b**).

In Figure 1, the carbonyl and amide II regions of H1, H2, H1Al, and H2Al samples (a) and the spectra of allantoin, β-CD, HP-β-CD, and Carbopol 934 (b) are presented.

The band at 1708 cm^−1^ in H1 and H2 samples is assigned to the stretching vibration of the carbonyl groups, redshifted by 10 cm^−1^ in comparison with the starting component (Carbopol 934), and it is correlated with the intermolecular hydrogen bonds among the carboxylic groups of Carbopol 934. This band is shifted to 1716 and 1712 cm^−1^ in H1Al and H2Al samples, respectively, and becomes broader due to the conformational changes of the polymeric chains during the neutralization of a part of the carboxylic groups with TEA and to the presence of allantoin involved in the H-bonding with Carbopol 934. Thus, the presence of the N-H^+^ is highlighted by the appearance of the band at 1560 cm^−1^ due to the asymmetric and symmetric deformation vibrations, which are blueshifted by 4–10 cm^−1^ in the spectra of H1Al and H2Al, respectively, also suggesting the interaction with allantoin molecules. The other bands attributed to the O-H stretch and N-H deformations at 3500 cm^−1^ and 1650 cm^−1^, as well as that assigned to C-O (1167 cm^−1^), are also shifted in the spectra of H1, H2, H1Al, and H2Al samples by 50 cm^−1^ as compared with the starting components (Figure 1b) confirming the neutralization of the carboxylic groups and the intermolecular interactions between Carbopol and allantoin [26]. The calculated hydrogen-bonded distances (*R*) and energy (*E_H_*) also revealed an increase in *E_H_* in the H1Al and H2Al, from 25.17 kJ (H1) and 24.45 kJ (H2) to 26.39 kJ (H1Al) and 25.75 kJ (H2Al), respectively, and a reducing in H bond distances from 2.760 Å (H1) and 2.763 Å (H2) to 2.757 Å (H1Al) and 2.759 Å (H2Al).

**Figure 2:**
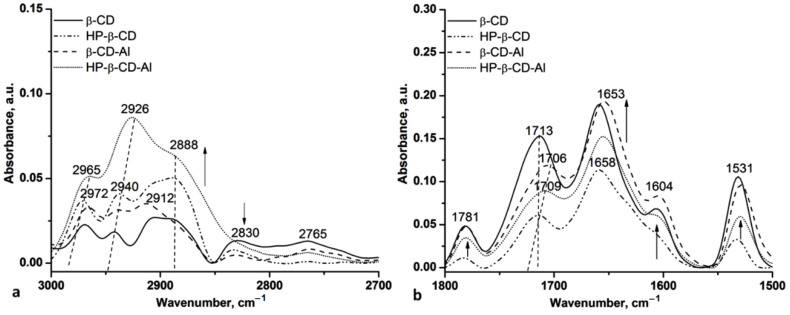
The C-H (**a**) and carbonyl, amide II (**b**) regions of β-CD, HP-β-CD and their inclusion complexes with allantoin (1:1).

The IR spectra of allantoin/β-CD and HP-β-CD (1:1) inclusion complexes also revealed the modification in the H bond distances and energy, confirming the inclusion of allantoin. Thus, the *E_H_* increases from 22.15 kJ (β-CD) to 25.60 kJ (β-CD-Al) and from 21.86 kJ (HP-β-CD) to 25.31 kJ (HP-β-CD-Al), while the *R* distances decrease by 0.01 Å in both cases (from 2.770 Å to 2.760 Å). The other spectral modifications can be seen in the C-H stretching and carbonyl regions (Figure 2).

For both inclusion complexes β-CD-Al and HP-β-CD-Al, all of the peaks of cyclodextrins appeared, and their spectra look almost similar to neat CDs indicating the formation of the inclusion complexes. These peaks were shifted, confirming the presence of allantoin in the complexes. Increases and decreases of intensities are remarked, too. These spectral changes indicate strong interactions between the guest-host molecules. The main modifications are marked in the figures by vertical dash lines and by arrows indicating the increasing/decreasing of the intensity and the band shifts.

For the C-H stretching regions, the bands of β-CD at 2965, 2940, and (2912 + 2888) cm^−1^ assigned to the asymmetric stretches of C-H bonds increase, while those assigned to the symmetric C-H stretches decrease. For HP-β-CD, one can observe the blueshift and the increasing intensity of the C-H asymmetric stretches from 2972 cm^−1^ to 2965 cm^−1^ and 2940 cm^−1^ to 2926 cm^−1^, while the position of the C-H symmetric stretches remains stable, only a slight decreasing is observed for the bands at 2830 and 2765 cm^−1^ (Figure 2a).

In the carbonyl and amide II stretching region, one can observe the bands at 1781 cm^−1^ assigned to the interaction of the non-neutralized carboxylic groups from gels with water leading to the formation of open dimers or oligomers [37] and the band at 1531 cm^−1^ assigned to the C-N groups from allantoin, overlapped with the N-H^+^ groups form TEA.

The inclusion of allantoin is also proved by the blueshifted of the C=O stretching vibration by 4–6 cm^−1^, from 1713 to 1706–1709 cm^−1^ in the spectra of the allantoin-based inclusion complexes, and by increasing the intensity and a slight blueshift by 5 cm^−1^ of the bands at 1658 and 1604 cm^−1^ assigned to the hydrogen bond interactions between NH_2_ and H-O groups.

**Figure 3:**
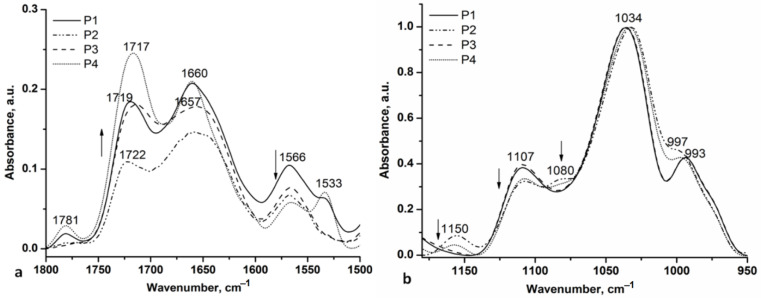
Carbonyl, amide II (**a**), and C-O-C (**b**) spectral regions for P1–P4 hydrogel samples.

In Figure 3, carbonyl, amide II (Figure 3a), and C-O-C (Figure 3b) spectral regions for P1–P4 hydrogel samples are presented.

The IR spectra of the P1–P4 samples in the 4000–2700 cm^−1^ are almost the same as the IR spectra of H1Al and H2Al, respectively, with the H bond distances and the H-bonding energy having very close values (24.59 kJ for P2 and P4; 26.39 kJ for P1 and P3), suggesting that the Carbopol 934 is the main component involved in the H bonds with the allantoin and allantoin-based inclusion complexes. In the carbonyl spectral range (Figure 3a), the main changes are assigned to the presence of the allantoin amide I (C=O···H-N−) and amide III stretches (N-H associated with C-N) at 1781 cm^−1^ and 1532 cm^−1^, respectively [38].

The other carbonyl bands are located as follows: P1 (1719 cm^−1^, 1660 cm^−1^, 1566 cm^−1^), P2 (1722 cm^−1^, 1653 cm^−1^, 1566 cm^−1^), P3 (1712 cm^−1^, 1654 cm^−1^, 1566 cm^−1^) and P4 (1717 cm^−1^, 1660 cm^−1^, 1566 cm^−1^). The changes in the intensity of the bands at 1717 cm^−1^ and 1660 cm^−1^ also suggest the modification in the conformation of the gel formulations components induced by the neutralization of the Carbopol 934 and the presence of the allantoin-based inclusion complexes. The spectral region 1200–950 cm^−1^ revealed the C-O and C-O-C stretches of the gel formulations. Thus, the bands at 1150 cm^−1^ (P2 and P4 samples), 1107 cm^−1^, and 993 cm^−1^ are characteristic of the C-O stretching vibration having a lower intensity in the gel formulations based on HP-β-CD. The C-O-C stretches for the same gel formulations (P2 and P4) evidenced two contributions at 1080 cm^−1^ and 1034 cm^−1^ and can be assigned to the different conformations of the HP-β-CD component (C-O-C stretching vibrations) and N-H^+^ rocking vibration.

### 3.2. Structural Characterization by ^1^H NMR Analysis

In ^1^H NMR spectra of H1, H2, H1Al, and H2Al, there are present the characteristic signals for Carbopol 934 at 2.35–1.7 ppm and glycerol at 3.97–3.53 ppm (Figure S1, Supplementary Materials). The presence of allantoin is confirmed by the peak at 5.39 ppm. In P1–P4 samples, these signals can be observed at 2.32–1.65 (carbopol fragment), 3.97–3.53 ppm (glycerol and β-CDs), and 5.1–5.2 ppm (anomeric protons of β-CDs) and allantoin at 5.39 ppm (Figure S2, Supplementary Materials).

### 3.3. Contact Angle Analysis

All the samples based on Carbopol exhibit low contact angle with water (less than 30) hence indicatinghighly hydrophilic surfaces (Table 2). The water contact angle values increase in the following order: P1 < P3 < P4 < H2Al < P2 < H1 < H2 < H1Al. In Table 3, the surface free energies and their components calculated for dried formulations are presented. In Table 4, the values of work of adhesion, free energy of hydration, Girifalco–Good’s interaction parameter, interfacial tension corresponding to water and to blood, and spreading parameter of dried formulations are tabulated.

The polarity of the biomaterial’s surface plays an important role in the interaction of the material with biological tissues, and its modification is reflected in the polar component of the surface free energy.The surface free energy of the material determines the cellular adhesion. It was found that highly hydrophilic substrates with high $\gamma_{sv}$ 40–58 mN/m and low contact angle and high $\gamma_{sv}^{p}/\gamma_{sv}\times 100$ show high capacity of bioaccumulation, and these substrates were most effective in testing bioadhesion [39]. Cell spreading is favored for $\gamma_{sv}^{p}$ values of polymer surfaces higher than 15 mN/m [40]. It is evident from Table 3 that the polar Lewis acid–base interactions ($\gamma_{sv}^{p}$) dominate the dispersive Lifshitz–van der Waals interactions ($\gamma_{sv}^{d}$). The electron acceptor $\gamma_{sv}^{+}$ (Lewis acid) component values are much higher than the electron donor $\gamma_{sv}^{-}$ (Lewis base) component values, which evidences the electron acceptor monopolar surfaces of the samples [41].

The adsorption of proteins is more extensive and less reversible at hydrophobic surfaces than at hydrophilic surfaces due to a greater extent of unfolding at hydrophobic surfaces leading to instantaneous protein adsorption, strong interfacial hydrophobic interactions, and displacement of vicinal water molecules from the unfavorable environment of the surface [42]. Initially, the adsorption of serum proteins occurs when biomaterials come into contact with blood. Then platelet adhesion occurs with activation of coagulation pathways and thrombus formation. It was reported in [43,44] that the interfacial free energy region should be at 1–3 mN/m to provide prolonged compatibility with blood. From Table 4, it is observed that the dried formulations except for P1 and H2Al ***γ_SL(blood)_*** values fall in this interval confirming their hemocompatible biomaterial qualities. The values of the spreading coefficient, *S_C_* < 0, are characteristic of a nonspreading system, and evidence of partial wetting conditions and the liquid mostly wets the solid surface if *θ* < 90°

It is reported in the literature [45] that when Δ*G_w_* < −113 mJm^−2^, the polymer is more hydrophilic (Table 4), while when Δ*G_w_* > −113 mJm^−2^ is more hydrophobic. The Δ*G_w_* values in Table 4 evidence the hydrophilic character of the dried formulations. The Δ*G_w_* values increase in the following order: P1 < P3 < P4 < H2Al < P2 < H1 < H2 < H1Al.

From Table 4, it can be observed that for water, $\Phi_{W}$ values are very close to unity revealing that the intermolecular (cohesive and adhesive) forces acting across the interface (regular) are similar. Work of adhesion values calculated for water, *W_a_* increases in the same order as hydrophilicity. Work of adhesion values calculated for blood are lower than those of water. This means blood adheres less than water to the biomaterial surface. When a blood vessel is injured, a coagulation cascade is initiated, which is very important for hemostasis. The calculated work of spreading of the red blood cells has positive values, while the work of the spreading of platelets has negative values, which denote that the contact of carbopol-based gels with blood will not allow easy adhesion of platelets, thus avoiding the appearance of thrombosis. Cellular adhesion influences the thrombogenicity and immunogenicity of the biomaterial and determines its blood compatibility [46].

### 3.4. Dynamic Vapor Sorption Analysis

Moisture transport in polymer systems is influenced by the presence of the holes in the polymer network and of the hydrogen bonding sites along the polymeric chain responsible for the attraction forces between the polymer and water molecules because two phenomena occur sequentially: water diffusion and polymer chain relaxation. Initially, water molecules fill up pores. Then, water molecules interact with the polymer, and the swelling occurs. Unbound moisture refers to the water molecules that are free to move through the holes or free volumes and does not cause swelling. The enclosed water molecules via hydrogen bonding sites disrupt the interchains hydrogen bonding determining swelling and plasticization of the polymer.

**Figure 4:**
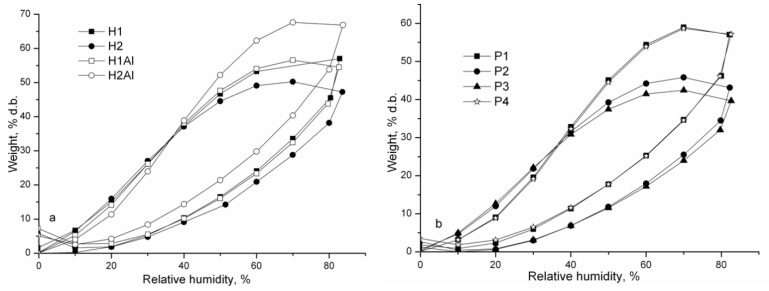
The sorption/desorption isotherms of studied hydrogels: a—H1, H2, H1Al, and H2Al samples; b—P1, P2, P3, and P4 samples.

In Figure 4, the sorption/desorption isotherms of studied hydrogels at different relative humidity (RH%) are shown. Adsorption and desorption isotherms express the dependency between the equilibrium water content of studied hydrogels at a distinct relative humidity value. All the samples exhibited a type III isotherm profile, characteristic of highly porous materials. A high moisture capacity of 55–67% for allantoin-based gels (H1Al and H2Al) and 39–57% for gels containing allantoin-based inclusion complexes was observed. At lower relative humidity steps (0–40%), a slight increase in moisture capacity occurred, while at higher relative humidity (40–80%), a rapid moisture increase as a result of gels swelling was observed (Figure 4). Hydrogen bonding dissociation influenced the moisture uptake, as well as the formation of water clusters inside the pores, also supported by the large hysteresis in the 40–80% RH [47].

The efficacy of water sorption is determined by the diffusion, sorption, and permeability coefficients. Their values are given in Table 5 and Table 6. First, the polymer adsorbed the water-enclosed molecules, followed by diffusion through the polymer. Segmental mobility of the polymer influences the diffusion coefficient. The swollen polymer chain determines the increased flexibility of the chain. The equilibrium stage is attained when further absorption of the water molecules becomesrestricted due to increased chemical potential.

Crank [33] and then Balik [48] have developed various methods of determining diffusion coefficients based on the second Fick’s equation, using the isotherm’s experimental data of sorption-desorption. The diffusion coefficient represents the rate of transfer of the enclosed molecules across the unit area of cross-section divided by the space gradient of concentration. In the case of Fickian behavior, the rate of diffusion is lower than the relaxation modes of the polymer matrix. Macromolecular chains fastly relax [33] such that the penetrant diffusion is slow and thus modeled using Fick’s law. Fick’s law predicts that, initially, the amount of absorbed water increases linearly with the square root of time and afterward slows down until saturation is attained.

From Table 5, it is observed that at short times (in the early stages of sorption, *Mt*/*M∞* < 0.5-first half sorption), the values of diffusion coefficient (D_1_) are lower than those (D_2_) corresponding to long times (in the later stages of sorption, *Mt*/*M∞* > 0.5, second half sorption) due to plasticizing effect of water. It was claimed that *D_1_* refers to water diffusion at the surface of the film, whereas *D_2_* refers to water diffusion atthe core of the film [49]. For polymers with enhanced hydrophilicity, the diffusion coefficient is low [50]. The decrease in diffusion coefficient has been explained byenhanced cluster size of the diffuse species, which become less movable [51].

The sorption coefficient refers to the strength of interactions between the polymer matrix and water molecules and describes the initial penetration and dispersal of the water molecules into the polymer matrix. The sorption coefficient increases in the following order: P3 < H2 < P2 < H1Al < P4 < P1 < H1 < H2Al. The permeability coefficient P_(1)_ values fall in the 1.17–2.73 × 10^−8^ range, whereas P_(2)_ values fall in 0.87–1.60 × 10^−7^.

### 3.5. Mechanical Tests

**Figure 5:**
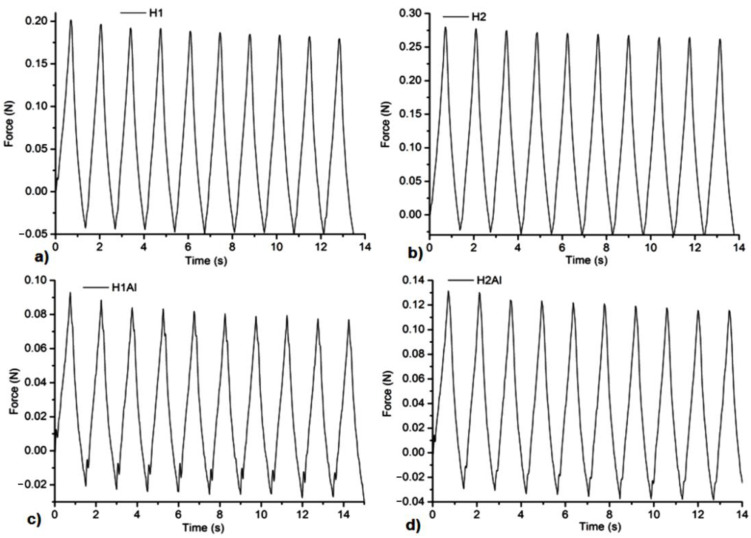
Compression tests of the samples at 20% compressive strains: (**a**)—H1, (**b**)—H2, (**c**)—H1Al, and (**d**)—H2Al.

**Figure 6:**
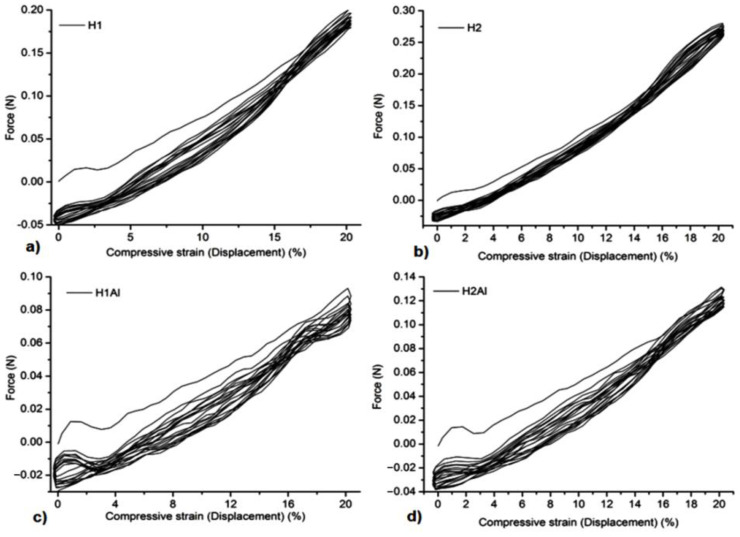
The dependence of Force/compressive strain% of the samples: (**a**)—H1, (**b**)—H2, (**c**)—H1Al, and (**d**)—H2Al.

**Figure 7:**
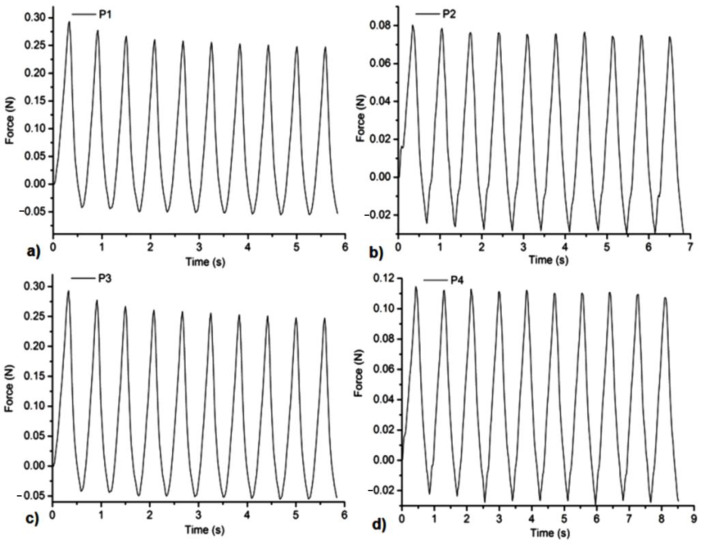
Compression tests of the samples at 20% compressive strains: (**a**)—P1, (**b**)—P2, (**c**)—P3, and (**d**)—P4.

**Figure 8:**
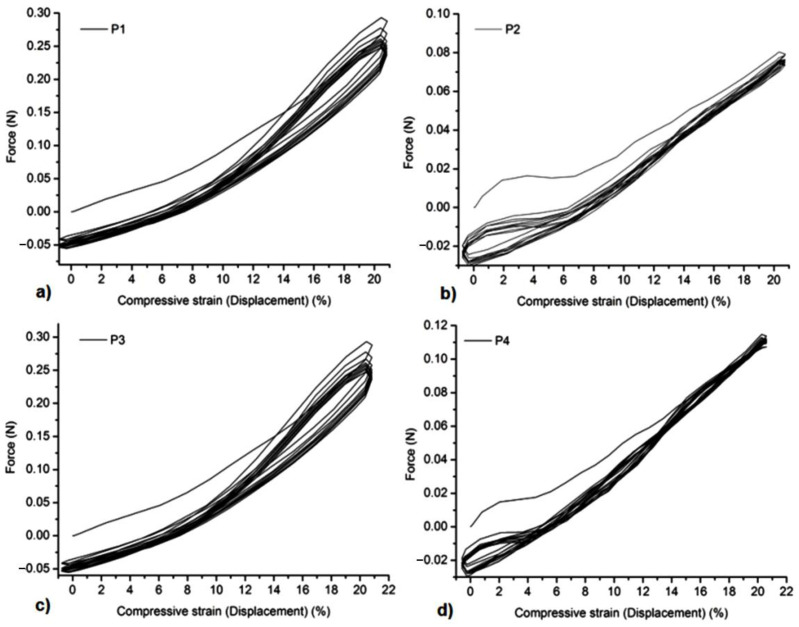
The dependence of Force/compressive strain % of the samples: (**a**)—P1, (**b**)—P2, (**c**)—P3, and (**d**)—P4.

All the samples showed a suitable resistance to compression forces and stability at several cycles of extension-compression without damage (Figure 5, Figure 6, Figure 7 and Figure 8). The concentration of Carbopol and the allantoin/inclusion complexes of allantoin proved to have an influence on the mechanical performance of the gels. Thus, the addition of allantoin led to an increase in viscoelasticity of the sample, a small hysteresis being observed for H1Al and H2Al in the force/compressive strain curves (Figure 6).Similar behavior was observed for P1–P4 samples, especially for those containing HP-βCD inclusion complexes with allantoin, highlighting the contribution of HP-βCD in the stabilization of the gels (Figure 8).

### 3.6. In Vitro Degradability of the Gels

**Figure 9:**
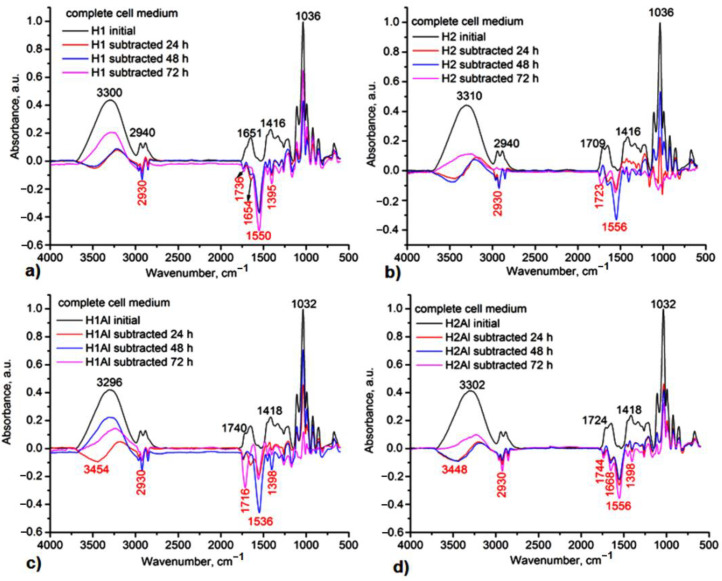
IR subtracted spectra of the H1-(**a**), H2-(**b**), H1Al-(**c**), and H2Al-(**d**) after 24 h, 48 h, and 72 h immersion in a complete cell medium.

**Figure 10:**
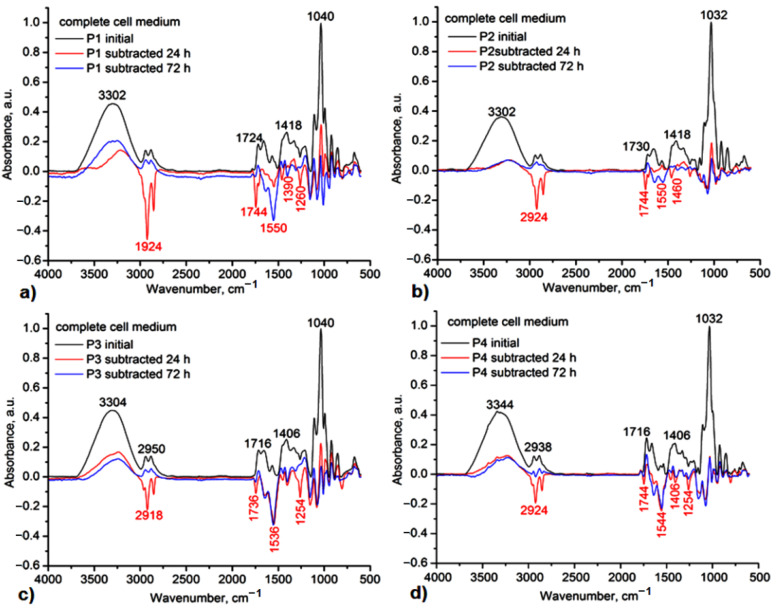
IR subtracted spectra of the P1-(**a**), P2-(**b**), P3-(**c**), and P4-(**d**) after 24 h, 48 h, and 72 h immersion in a complete cell medium.

All gels proved suitable stability under mild conditions of pH 7.4, mimicking the body’s physiological conditions. In Figure 9 and Figure 10, one can observe the subtracted IR spectra of the gels. The main degradation products identified by IR subtracted spectra between the initial spectrum and the different ones after hydrolysis at 24 h, 48 h, and 72 h were found in small fragments from Carbopol and glycerol for H1 and H2 samples, to which allantoin is added as a release product from the gels’ networks in H1Al and H2Al samples (Figure 9). The negative peaks confirming these polymer/allantoin products are those at 3454, 2930, 1716–1744, 1668, 1556, and 1398 cm^−1^ assigned to the stretching vibrations of O-H groups, aliphatic C-H, and COOH/COOC and C-O-C groups, respectively. In biological media, in vivo, these fragments could also occur as a result of the native immune response of the organism. This response is mainly mediated by the activity of neutrophils and macrophages, which can generate strong oxidants by involving specific enzymatic reactions [52]. The hydrolysis products for P1–P4 samples consisted of small fragments of Carbopol and glycerol, βCDs, and allantoin from the inclusion complexes. As it can be observed in the IR subtracted spectra after 24 h of immersion in a complete cell medium, the main product is carbopol, while allantoin can be identified after 72 h of hydrolysis, suggesting a relatively low rate of release from the inclusion complexes (Figure 10). In order to confirm its presence, the UV-vis spectra of the media after immersion of these gels have been registered, and allantoin was identified by its characteristic absorption maximum at 224 nm.

### 3.7. Cell Culture Tests

**Figure 11:**
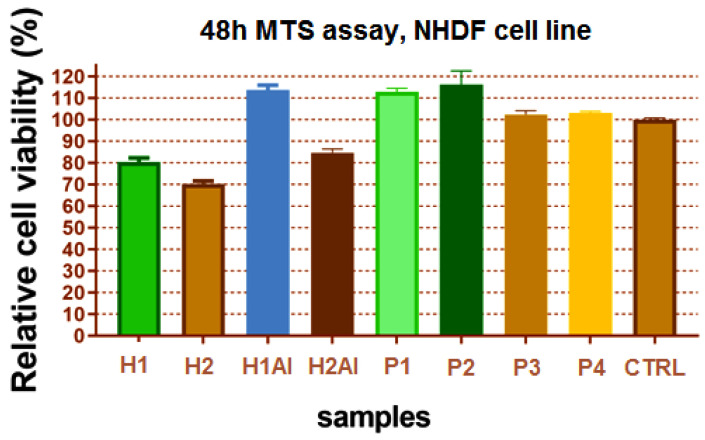
The cell viability on the NHDF cell line of the studied gels.

The result of the cell viability revealed suitable biocompatibility of the gels containing allantoin and allantoin/β-CDs inclusion complexes (Figure 11). Lower cytotoxicity was evidenced for H1 and H2 gels (80% and 70%, respectively), which is due to the acidity of the samples, and the presence of the polyacrylic acid-free groups in contact with the cell medium being responsible for this behavior. In these conditions, the contribution of allantoin and allantoin/β-CDs inclusion complexes proved to be beneficial for cell viability.

**Figure 12:**
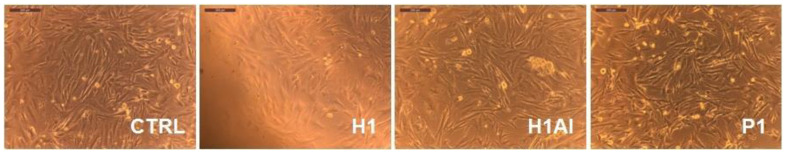
The appearance of the fibroblasts after 48 h incubation with gels: H1, H1Al and P1 in comparison with the control (CTRL) sample.

Moreover, the microscope images of the NHDF cells after 48 h incubation with gels revealed suitable biocompatibility with cells, with minor changes in fibroblasts culture after H1 incubation. This supports the ability of these gels to act as matrices for cells. In a cell medium, the swelling of the gel can favor cell adhesion, promoting the intrinsic mechanisms of cellular regeneration (Figure 12).

### 3.8. Bioadhesion and Mucoadhesion Tests

The bio- and mucoadhesivity of the allantoin and its β-cyclodextrin inclusion complexes-loaded gels were evaluated in vitro by direct determination of the detachment force and the work of adhesion on different synthetic and biological substrates, cellulose membrane, a typical model for mimicking the cell membrane and porcine stomach mucosa, taking into account the gastroprotective role of allantoin-based formulation in the treatment of gastric diseases. Thus, different nanocomposites loading allantoin (silica nanoparticles, liposomes, or lipid particles) have been developed and used in biocompatible polymeric matrices for biomedical administration and tissue healing. The mucoadhesivity of such formulations was found to be dependent on the polymeric matrix-mucin binding affinity (based on hydrogen bonding or electrostatic interactions), which is the key factor influencing the gastric residence time and the cellular uptake of allantoin [53].

**Figure 13:**
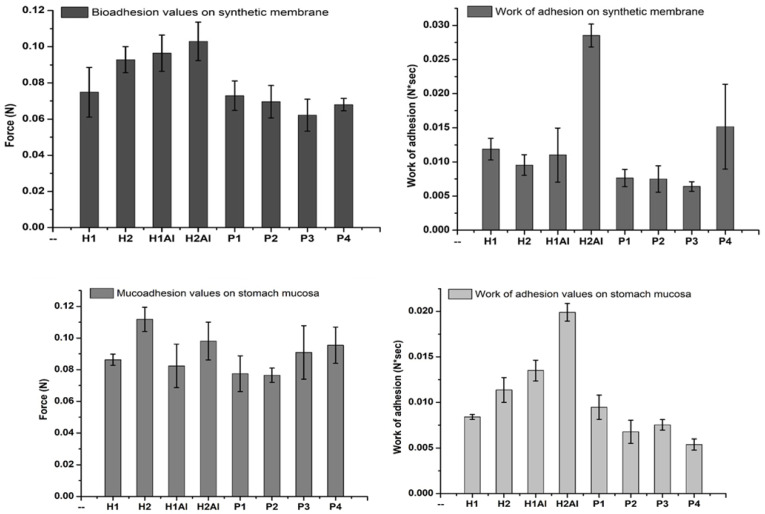
Bioadhesionand mucoadhesiontests for studied formulations.

The bioadhesion and mucoadhesion evaluation of the allantoin-based gelformulations are depicted in Figure 13. It is evident a great variation in the bioadhesive/mucoadhesive strengths due to the Carbopol 934 and allantoin or its β-cyclodextrin inclusion complexes. All the gel formulations showed high values of the detachment force on both substrates (cellulose membrane and stomach mucosa). The encapsulation of allantoin in the gels led to increased values of the detachment force as compared with the use of inclusion complexes. The lower values can be explained by the strong connection of the inclusion complexes within the gel network. There is a strong dependence of these values on the water sorption capacity and wettability of the samples, which are higher for H1Al and H2Al than the corresponding samples, including β-cyclodextrins complexes with allantoin. The wettability of the samples is considered the first mechanism of adhesion, which occurs in the contact phase, followed by the complex mechanisms of adsorption and diffusion involving strong interaction between polymeric chains and mucus constituents in the consolidation phase. One can observe that the detachment force increased with the Carbopol and HP-β-cyclodextrin concentrations in gels (P3 and P4). A higher content of polymer increases the surface area and more polymer chains for interpenetration with mucin, and, therefore, the bioadhesive/mucoadhesive strength is enhanced. The chosen polymer’s mobility must be high in order to assure the interpenetration of the hydrogel into the oral/stomach mucosa and is considered to determine the kinetic part of the adhesion process. The values of the work of adhesion are almost the same for the gels, including the allantoin-β-cyclodextrins inclusion complexes, proving that similar mechanisms are involved in the contact and consolidation steps of adhesion with stomach mucosa. The highest values for the detachment force and work of adhesion have been obtained for the polymeric matrix and the gel formulation embedding allantoin because of its humectant and moisturizer qualities [54]. Moreover, higher hydration of the gels would lead to a rapid local drug release, while a lower wettability will ensure a prolonged release, thus improving the drug bioavailability. In addition, one can observe a dependence of the work of adhesion on the pH values. At pH 7.4, the polymeric matrix based on Carbopol is swelling higher than at pH 2.6, which also explains the lower values of work of adhesion on stomach mucosa.

### 3.9. Antimicrobial Susceptibility Tests

Allantoin is an imidazolidine-type alkaloid that manifests antibacterial and antifungal activities [54,55] against the tested microorganisms presented in Table 7. Interaction effects were identified for Carbopol on antimicrobial activity of methyl *para*-hydroxybenzoate against some Gram-negative and Gram-positive bacteria and yeast [56]. Glycerol manifests bactericidal activity as well [57].

The diameters of the inhibition zones (in mm) corresponding to the tested hydrogels are presented in Table 7. The results are given as means ± SD.

**Figure 14:**
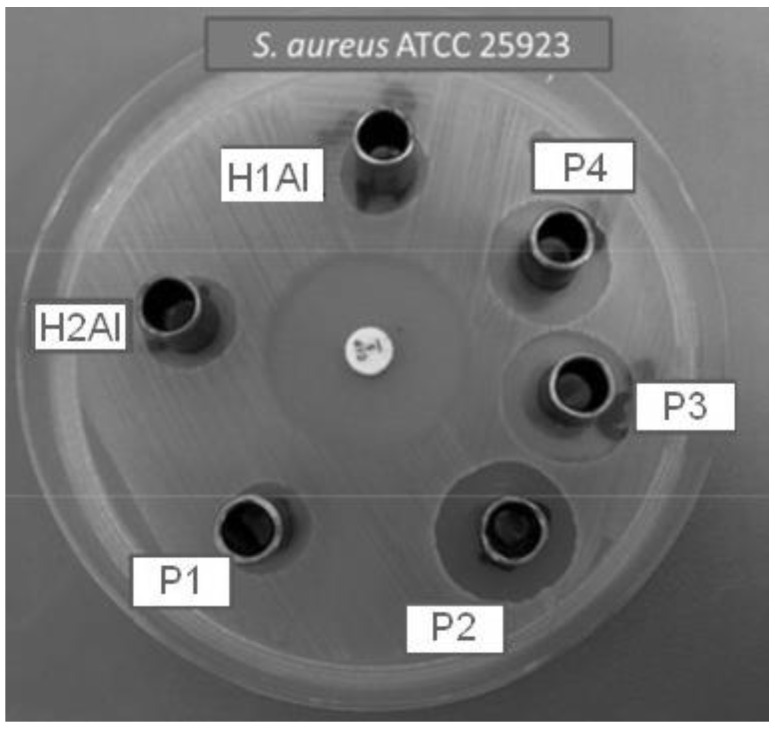
Antibacterial activity of tested compounds (H1–H2 and P1–P4) against *S. aureus* ATCC 25923.

The inhibition zones of the studied samples against *S. aureus* ATCC 25923 are shown in Figure 14.

The tested hydrogels manifest suitable antibacterial activity against *S. aureus* ATCC 25923. Among P1–P4 compounds, P2, P3, and P4 have a suitable activity against Gram-positive bacteria and are better than the samples without inclusion complexes, H1Al and H2Al. All the tested samples did not have any activity against Gram-negative bacteria (*E. coli* ATCC 25922 and *P. aeruginosa* ATCC 27853). The hydrogels without inclusion complexes H1Al and H2Al manifest a reduced antifungal activity in comparison with P3 and P4 samples.

The antimicrobial spectrum of these hydrogels may be explained by the differences in their qualitative and quantitative chemical composition.

The neutralized aqueous solution of Carbopol 934 exhibited no microbial activity on the studied strains. In combination with allantoin and allantoin-β-CD inclusion complexes, the concentrations of Carbopol 934 of 0.5% and 1% are sufficient to produce a considerable increase in viscosity and the stability of gels. Thus, the carbopol antimicrobial synergism appears as a simple mechanism to provoke a strong inhibition effect on the selected strains. The concentration of Carbopol 934 in the mentioned samples H2Al, P3, and P4 is the same, 1%. This mechanism also has been reported for different Carbopol 940/parahydroxybenzoate pharmaceutical formulations. Such formulation exhibited an interesting synergism against *E. coli* and *P. aeruginosa*, as well as a decrease in antimicrobial activity at higher concentrations of polymer, especially against *S. aureus* and *C. albicans* [56]. Recent studies revealed that this mechanism can be described by the disorder created in the microbial membrane in contact with the polysaccharides-based composite membranes. The electrostatic interactions are involved in the adhesion process with the microbial membrane. The complex formation between the polymers and membrane cells disrupts its functionality and functions, promoting the cell death of the microbial strains [58]. The intrinsic porosity of the polymeric materials self-assembled in nanospheres proved to be another strategy to promote antibacterial activity. It was found that the association of such polymeric nanoparticles with some imidazolium-based ionic liquids will be beneficial for antimicrobial applications, especially in food packaging and storage, water purification, disinfection of medical devices, etc. [59]. Moreover, poly(*a*-L-glutamic acid)-based nanomaterials were found as a new route to promote the drug encapsulation and release at a trigger location. These materials were successfully applied as antimicrobial agents and in cancer therapy as drugs and protein delivery systems [60].

## 4. Conclusions

In the present work, mucoadhesive gel formulations based on Carbopol 934 and allantoin-based β-cyclodextrins (1:1) inclusion complexes were prepared according to a default protocol and characterized in terms of structural analysis, contact angle measurements, water vapor sorption capacity, mechanical performance, biocompatibility, in vitro degradability, bio-/mucoadhesivity, and antimicrobial activity. IR and ^1^H NMR spectroscopies were used to evidence the main structural characteristic of the gel formulations with allantoin and allantoin-based inclusion complexes. It was observed that increased values of the H bonds energy and H bonds strength for all gels having allantoin and allantoin-based inclusion complexes in the structure. Based on these interactions, a self-healing behavior of the gels in different media can be developed. The spectral changes also indicated strong interactions between the guest-host molecules for allantoin-cyclodextrins (1:1) inclusion complexes. All the gel formulations evidenced a hydrophilic character proved by the low water contact angle and water sorption capacity values. The values were found lower when allantoin-based inclusion complexes were added, highlighting the more pronounced hydrophilicity. Carbopol and allantoin favored increased values of force and work of adhesion, the highest values being observed for the H2Al sample on both synthetic (cellulose) membrane and stomach mucosa. All studied hydrogels evidenced suitable biocompatibility on NHDF cell culture and stability in cell medium, as well as antibacterial activity against *S. Aureus*, but a pronounced activity was found for the gel formulations with inclusion complexes, especially against *S. Aureus* and *C. Albicans*. In addition to these important characteristics, the studied formulations proved suitableinterfacial free energy values suggesting their prolonged compatibility with blood, which makes them suitable as scaffolds for tissue engineering applications such as wound dressing and gastric protective gels.

## Figures and Tables

**Table 1:** Composition of the bioadhesive gels.

Sample	Materials						
	Carbopol 934	Glycerol	TEA (Drops)	Allantoin %	β-CD	HP-β-CD	Distilled Water q.s.
H1	0.5	10	3	-	-	-	100
H2	1	10	5	-	-	-	100
H1Al	0.5	10	3	0.2	-	-	100
H2Al	1	10	5	0.2	-	-	100
P1	0.5	10	3	0.2	1.44	-	100
P2	0.5	10	3	0.2	-	1.75	100
P3	1	10	5	0.2	1.44	-	100
P4	1	10	5	0.2	-	1.75	100

**Table 2:** Mean static contact angles determined by the sessile drop method.

Samples	Contact Angle (°)		
	Water	Formamide	Diiodomethane
H1	26.60	23.47	89.03
H2	29.94	28.09	91.25
P1	23.12	33.16	102.23
P2	26.49	25.82	79.88
P3	24.35	36.81	85.75
P4	25.75	30.32	89.62
H1Al	30.77	26.84	80.69
H2Al	26.13	31.50	99.54

**Table 3:** Surface free energies and their components of dried formulations in mN/m.

Samples	$\gamma_{sv}^{d}$	$\gamma_{sv}^{+}$	$10^{6}\times \gamma_{sv}^{-}$	$\gamma_{sv}^{p}$	$\gamma_{sv}^{p}/\gamma_{sv}\times 100$	$\gamma_{sv}$	Δ*G_w_*
H1	13.13	15.81	9.61	53.08	80.14	66.21	−138
H2	12.15	104.65	4.00	52.34	80.17	64.49	−136
P1	7.89	126.27	9.00	63.18	88.91	71.07	−140
P2	17.56	95.83	25.00	47.88	73.17	65.44	−138
P3	14.65	104.76	0.64	52.39	78.15	67.04	−139
P4	12.87	107.89	9.00	53.91	80.73	66.78	−138
H1Al	17.14	91.60	6.25	45.83	72.78	62.97	−135
H2Al	8.84	119.47	0.04	59.74	87.11	68.58	−138

**Table 4:** The values of work of adhesion, free energy of hydration, Girifalco–Good’s interaction parameter, interfacial tension corresponding to water and to blood, and spreading parameter of dried formulations.

Samples	*W_a_*, mN/m	*W_a(blood)_*, mN/m	Δ*G_w_*, mN/m	$\Phi_{W}$	$S_{C}$, mN/m	$\gamma_{sl}$, mN/m	$\gamma_{sl(blood)}$, mN/m	*W_s/rbc_*, mN/m	*W_s/p_*, mN/m
H1	138	112	−137.89	0.99	−7.89	1.30	1.67	42	−143
H2	136	111	−135.88	0.99	−9.72	1.41	1.48	110	−111
P1	140	115	−139.76	0.97	−5.86	4.11	3.99	115	−119
P2	138	111	−137.96	0.99	−7.64	0.28	1.51	112	−100
P3	139	113	−139.13	0.99	−6.48	0.72	1.70	113	−104
P4	138	112	−138.37	0.99	−7.23	1.21	1.79	113	−108
H1Al	135	109	−135.35	0.99	−10.25	0.42	1.18	108	−102
H2Al	138	113	−138.16	0.98	−7.44	3.22	3.04	113	−118

**Table 5:** Diffusion coefficients determined from experimental data of studied hydrogel samples.

Sample	*K*_1_*, *M_t_*/*M_∞_* < 0.5	*K*_2_*, *M_t_*/*M_∞_* > 0.5	*l* (cm)	*D*_1_ = *K*_1_π*l*^2^/16 (cm^2^/s)	*D*_2_ = −*K*_2_*l*^2^/*π*^2^ (cm^2^/s)
H1	1.89 × 10^−5^	−0.00027583	0.1	3.71 × 10^−8^	2.80 × 10^−7^
H2	1.53 × 10^−5^	−0.00022095	0.1	3.01 × 10^−8^	2.24 × 10^−7^
H1Al	1.70 × 10^−5^	−0.00021338	0.1	3.35 × 10^−8^	2.16 × 10^−7^
H2Al	1.93 × 10^−5^	−0.0002072	0.1	3.79 × 10^−8^	2.10 × 10^−7^
P1	2.58 × 10^−5^	−0.00026206	0.1	5.06 × 10^−8^	2.66 × 10^−7^
P2	2.31 × 10^−5^	−0.00028869	0.1	4.53 × 10^−8^	2.93 × 10^−7^
P3	2.10 × 10^−5^	−0.00027841	0.1	4.13 × 10^−8^	2.82 × 10^−7^
P4	2.18 × 10^−5^	−0.00025906	0.1	4.29 × 10^−8^	2.63 × 10^−7^

* *K_1_ is slope of linear regression between (t − t_R_) and (M_t_/M_∞_)^2^ for (t − t_R_) ≥ 0 and (M_t_/M_∞_)^2^ < 0.2 where t_R_ is time correlation (minus intercept/slope) for M_t_/M_∞_ = 0; K_2_ is slope of linear regression between t and ln(1 − M_t_/M_∞_) for −1.2 > ln > −3.0*.

**Table 6:** Sorption and permeability coefficients (cm^2^s^−1^), uptake weight at RH 80%.

Sample	S	P_(1)_ × 10^8^	P_(2)_ × 10^7^	Uptake Weight at RH 80%, % d.b.
H1	0.57	2.11	1.60	45.40
H2	0.39	1.17	0.87	32.30
H1Al	0.44	1.47	0.95	36.65
H2Al	0.59	2.24	1.24	48.71
P1	0.54	2.73	1.44	44.40
P2	0.41	1.86	1.20	33.61
P3	0.36	1.49	1.02	29.5
P4	0.52	2.23	1.37	42.98

**Table 7:** Antibacterial and antifungal activities of the tested hydrogels.

Samples	Diameter of Inhibition Zones (mm)			
	*S. aureus* ATCC25923	*E. coli* ATCC 25922	*P. aeruginosa* ATCC27853	*C. albicans* ATCC10231
H1Al	12.1 ± 0.05	0	0	11.5 ± 0.50
H2Al	13.0 ± 0.00	0	0	12.5 ± 0.50
P1	11.0 ± 0.00	0	0	0
P2	17.0 ± 0.00	0	0	0
P3	16.3 ± 0.57	0	0	16.3 ± 0.57
P4	16.0 ± 0.00	0	0	19.0 ± 0.00
Ciprofloxacin (5 µg/disc)	27.7 ± 0.06	32.0 ± 0.00	32.0 ± 0.00	NT*
Fluconazol (25 µg/disc)	NT*	NT*	NT*	30.0 ± 0.00
Voriconazol (1 µg/disc)	NT*	NT*	NT*	29.5 ± 0.50

NT*—not tested.

## Data Availability

The data presented in this study are available on request from the corresponding authors.

## Supplementary Materials

The following supporting information can be downloaded at: https://www.mdpi.com/article/10.3390/gels8070416/s1, Figure S1. ^1^H NMR spectra of: (a)-H1, (b)-H2, (c)-H1Al, (d)-H2Al; Figure S2. ^1^H NMR spectra of: (a)-P1, (b)-P2, (c)-P3, (d)-P4.
