# Supplementary material for: Mucoadhesive and Antimicrobial Allantoin/β Cyclodextrins-Loaded Carbopol Gels as Scaffolds for Regenerative Medicine
Source: Gels. 2022 Jul 2;8(7):416. doi: 10.3390/gels8070416 (PMC9320337; doi:10.3390/gels8070416)
Supplement: Supplementary file 1 [file gels-08-00416-s001.zip › gels-1770434-supplementary.pdf]

Supplementary Materials

Mucoadhesive and Antimicrobial Allantoin/β cyclodextrins-loaded Carbopol Gels as Scaffolds for Regenerative Medicine

Daniela Filip[1], DoinaMacocinschi[1*], Mirela-Fernanda Zaltariov[1*], Carmen Anatolia Gafitanu[2], Cristina Gabriela Tuchilus[3], Adrian Bele[1], Bianca-Iulia Ciubotaru[1], Elena Stoleru[1] and Alexandra Bargan[1]

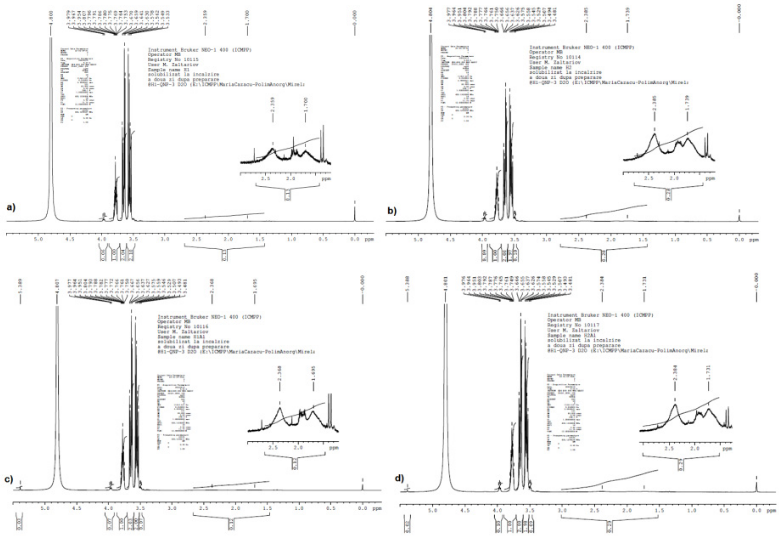

Figure S1. ^{1}H NMR spectra of: a)-H1, b)-H2, c)-H1Al, d)-H2Al

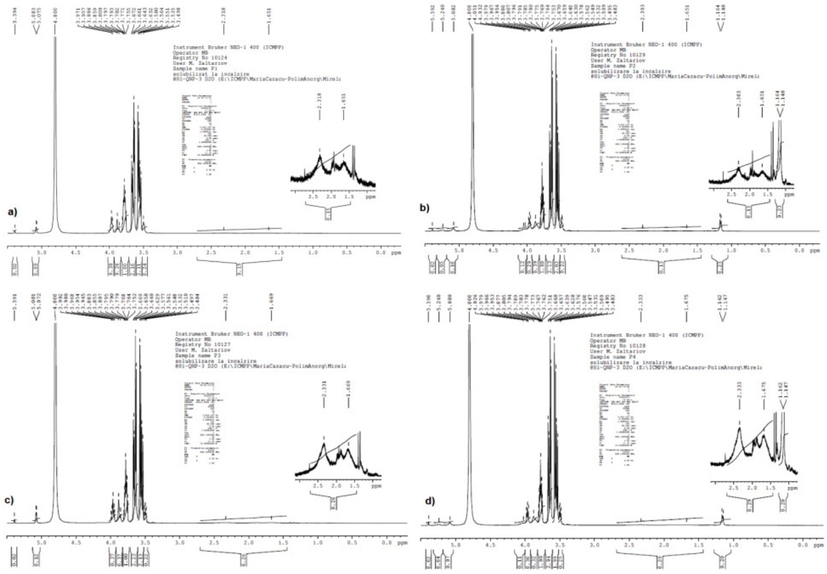

Figure S2. ^{1}H NMR spectra of: a)-P1, b)-P2, c)-P3, d)-P4
